# An Inhibitory Antibody Blocks Interactions between Components of the Malarial Invasion Machinery

**DOI:** 10.1371/journal.ppat.1000273

**Published:** 2009-01-23

**Authors:** Christine R. Collins, Chrislaine Withers-Martinez, Fiona Hackett, Michael J. Blackman

**Affiliations:** Division of Parasitology, National Institute for Medical Research, Mill Hill, London, United Kingdom; Institut Pasteur, France

## Abstract

Host cell invasion by apicomplexan pathogens such as the malaria parasite *Plasmodium* spp. and *Toxoplasma gondii* involves discharge of proteins from secretory organelles called micronemes and rhoptries. In *Toxoplasma* a protein complex comprising the microneme apical membrane antigen 1 (AMA1), two rhoptry neck proteins, and a protein called Ts4705, localises to the moving junction, a region of close apposition between parasite and host cell during invasion. Antibodies against AMA1 prevent invasion and are protective *in vivo*, and so AMA1 is of widespread interest as a malaria vaccine candidate. Here we report that the AMA1 complex identified in *Toxoplasma* is conserved in *Plasmodium falciparum*. We demonstrate that the invasion-inhibitory monoclonal antibody (mAb) 4G2, which recognises *P. falciparum* AMA1 (PfAMA1), cannot bind when PfAMA1 is in a complex with its partner proteins. We further show that a single completely conserved PfAMA1 residue, Tyr251, lying within a conserved hydrophobic groove adjacent to the mAb 4G2 epitope, is required for complex formation. We propose that mAb 4G2 inhibits invasion by preventing PfAMA1 from interacting with other components of the invasion complex. Our findings should aid the rational design of subunit malaria vaccines based on PfAMA1.

## Author Summary

Malaria is caused by a singe-celled parasite that invades and grows within red blood cells. Many available antimalarial drugs are increasingly ineffective, and there is no vaccine. Certain malarial proteins induce protective antibody responses that prevent red cell invasion. This study focuses on the mechanism by which an antibody called 4G2, specific for a parasite protein called AMA1, prevents invasion. Just before invasion, AMA1 is discharged onto the parasite surface, where it interacts with other parasite proteins at a transient structure called the ‘moving junction’, through which the parasite moves as it enters the cell. We have identified all the components of this protein complex in *Plasmodium falciparum*, the causative agent of the most dangerous form of malaria. We show that whereas 4G2 can bind to free AMA1, it cannot bind when AMA1 is in a complex with its partner proteins. This suggests that antibody binding just before invasion prevents assembly of the complex. In support of this, we show that amino acids within a surface-located ‘hydrophobic trough’ on AMA1, very close to the site recognised by 4G2, are involved in formation of the AMA1 complex. These findings suggest ways in which to optimally design antimalarial vaccines based on AMA1.

## Introduction

Malaria is a global problem, affecting many of the world's poorest nations. Around 40% of the world's population is at risk from the disease with over 500 million clinical cases yearly, and malaria is a major cause of mortality in children under five. Of particular concern, the emergence of insecticide-resistant mosquito vectors and multi-drug resistant parasites has contributed to resurgences of the disease in areas where it was previously under control. Malaria is caused by protozoan parasites of the genus *Plasmodium*, with the most severe disease being the result of infection with *P. falciparum*. The malaria merozoite invades erythrocytes and undergoes rounds of asexual replication (schizogony) to generate a schizont containing multiple daughter merozoites. Upon schizont rupture, the merozoites are released to invade new erythrocytes. Repetitive cycles of invasion, replication and schizont rupture are responsible for the clinical symptoms of the disease. The development of a malaria vaccine and the identification of new parasite targets for chemotherapeutic intervention are important ways forward in combating the disease.

Erythrocyte invasion by the merozoite proceeds in several rapidly consecutive steps. Initial low affinity binding to the host cell is followed by reorientation of the parasite so that its apical prominence is in close apposition with the host cell surface, formation of an intimate, electron-dense point of contact or ‘junction’ between parasite and host cell, then movement of the parasite into the parasitophorous vacuole, concomitant with translocation of this ‘moving junction’ across the parasite surface. Invasion is orchestrated by proteins released from merozoite apical secretory organelles called micronemes and rhoptries. Apical membrane antigen 1 (AMA1) is a type I integral membrane protein that plays an essential role in invasion. In *P. falciparum* it is synthesised during schizogony as an 83 kDa precursor called PfAMA1_83_ [1] and targeted to micronemes [2],[3]. Prior to invasion it is proteolytically processed to a 66 kDa form (PfAMA1_66_) that translocates onto the merozoite surface [1],[4] from where it is eventually shed during invasion by a membrane-bound subtilisin-like protease called PfSUB2 [5]–[7]. Homologues of AMA1 are present in all species of *Plasmodium* and in all other apicomplexan genera examined [8]–[11]. In *P. falciparum* [12], *Toxoplasma gondii* [13], and recently in the rodent malaria *P. yoelii* [14], AMA1 has been shown to interact with the essential rhoptry neck protein RON4. In *T. gondii* the AMA1/RON4 complex associates with the moving junction during invasion [12],[15]. Two additional AMA1-associated proteins (AAPs) have been identified in *T. gondii*; these are the rhoptry neck protein RON2 and a previously uncharacterised protein called Ts4705. Although homologues of both proteins exist in *Plasmodium*, evidence to suggest any association between these molecules and the PfAMA1-PfRON4 complex in *P. falciparum* is lacking.

The AMA1 ectodomain comprises three disulphide-constrained domains [16]–[18]. Immunisation with AMA1 or recombinant fragments of it can protect against blood-stage malarial infection, and antibodies against AMA1 inhibit erythrocyte invasion. As a result, AMA1 is of widespread interest as a malaria vaccine candidate (recently reviewed by Remarque et al. [19]). As with many malarial antigens, PfAMA1 exhibits significant polymorphism [20],[21], believed to facilitate evasion of inhibitory antibodies. The mechanism(s) of action of invasion-inhibitory anti-AMA1 antibodies has been a subject of considerable interest, but remains unclear. Whilst there is evidence that some antibodies may act by inhibiting translocation of AMA1 across the merozoite surface and its subsequent shedding by PfSUB2 [22], an alternative possibility is that antibodies may bind regions of the AMA1 ectodomain that are functionally important. Monoclonal antibody (mAb) 4G2 is a potent inhibitor of erythrocyte invasion by all strains of *P. falciparum* [23]. We previously demonstrated that the residues recognised by mAb 4G2 lie exclusively within the base of a loop in domain II of the PfAMA1 ectodomain [17],[24]. While the bulk of this loop extends across the non-polymorphic face of domain I, two residues of the loop (Lys357 and Phe367) form part of a conspicuous, surface-exposed, conserved hydrophobic trough in domain I that is surrounded by polymorphic residues [18]. No polymorphic residues have been identified within the domain II loop itself, suggesting that variation within this region of the molecule is functionally constrained [20]. If the domain II loop and adjacent residues are of functional importance, mAb 4G2 may act by blocking this function.

Here we provide evidence that this is indeed the case. We first demonstrate that the homologues of all three *Toxoplasma* AAPs are expressed in *P. falciparum* and interact specifically with PfAMA1. We then show that, in contrast to polyclonal antibodies against the PfAMA1 ectodomain, mAb 4G2 can bind PfAMA1 only when it is not in a complex with AAPs. Using transgenic expression of PfAMA1 mutants in the parasite we demonstrate that substitution of selected residues close to the 4G2 epitope and within the hydrophobic trough of PfAMA1, abolishes binding to RON4 and the other AAPs. Our findings suggest that mAb 4G2 inhibits invasion by blocking the formation of a functional complex between PfAMA1 and other components of the moving junction.

## Results

### PfAMA1 forms a complex with three AAPs

**Figure 1:**
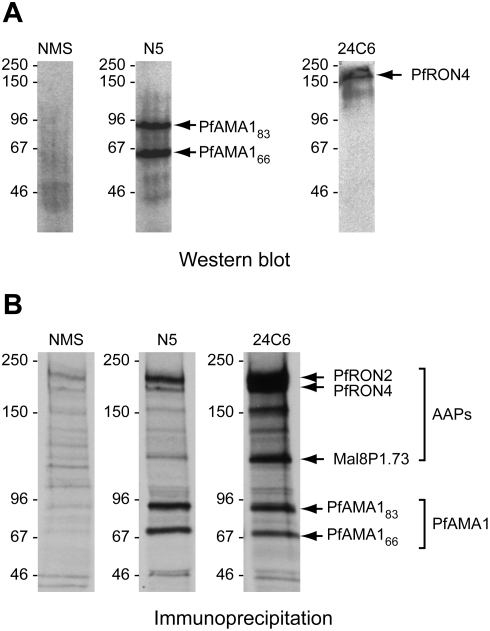
The PfAMA1 complex comprises PfAMA1 plus three AAPs. (A). Western blot of schizont extracts, demonstrating the specificity of binding of anti-PfAMA1 serum N5 and mAb 24C6 for PfAMA1 and PfRON4 respectively. Signals of the expected sizes were obtained, with no evidence for cross-reactivity. (B). Anti-PfAMA1 serum N5 and mAb 24C6 both precipitate PfRON4 and PfAMA1, plus two additional proteins of ∼190 kDa and 110 kDa. These were identified by mass spectrometric tryptic peptide mapping as PfRON2 and Mal8P1.73 respectively (Tables S1, S2, S3). IPs were from extracts of biosynthetically radiolabelled schizonts. Note that both the mature and processed forms of PfAMA1 were co-precipitated by mAb 24C6, suggesting that PfRON4 is able to interact with both forms of PfAMA1. NMS, normal mouse serum. Positions of molecular mass marker proteins are indicated (kDa).

In both *Toxoplasma* and *Plasmodium*, AMA1 interacts with the rhoptry neck protein RON4 [12]–[14]. Two additional AAPs, TgRON2 and Ts4705, were identified in *Toxoplasma*, both of which have putative homologues in *P. falciparum* [12],[13],[15]. To fully characterise the PfAMA1-PfRON4 complex, we used the anti-PfAMA1 polyclonal serum N5 [24] and the anti-PfRON4 mAb 24C6 [25], both of which are highly specific for their cognate antigens on Western blots (Figure 1A). When used to immunoprecipitate (IP) from parasite extracts, both antibodies co-precipitated the reciprocal protein(s) as expected, plus in both cases two species at ∼190 kDa and 110 kDa (Figure 1B). These latter proteins were unequivocally identified by matrix-assisted laser desorption/ionization time-of-flight (MALDI-TOF) analysis of tryptic peptide digests as products of *P. falciparum* genes Pf14_0495 (the homologue of TgRON2, hereafter referred to as PfRON2) and Mal8P1.73 (the *P. falciparum* homologue of Ts4705) respectively (Tables S1, S2, S3). These findings confirm that the invasion complex previously identified in *Toxoplasma* is conserved in its entirety in *P. falciparum*.

### The invasion-inhibitory mAb 4G2 cannot bind the intact PfAMA1-AAP complex

**Figure 2:**
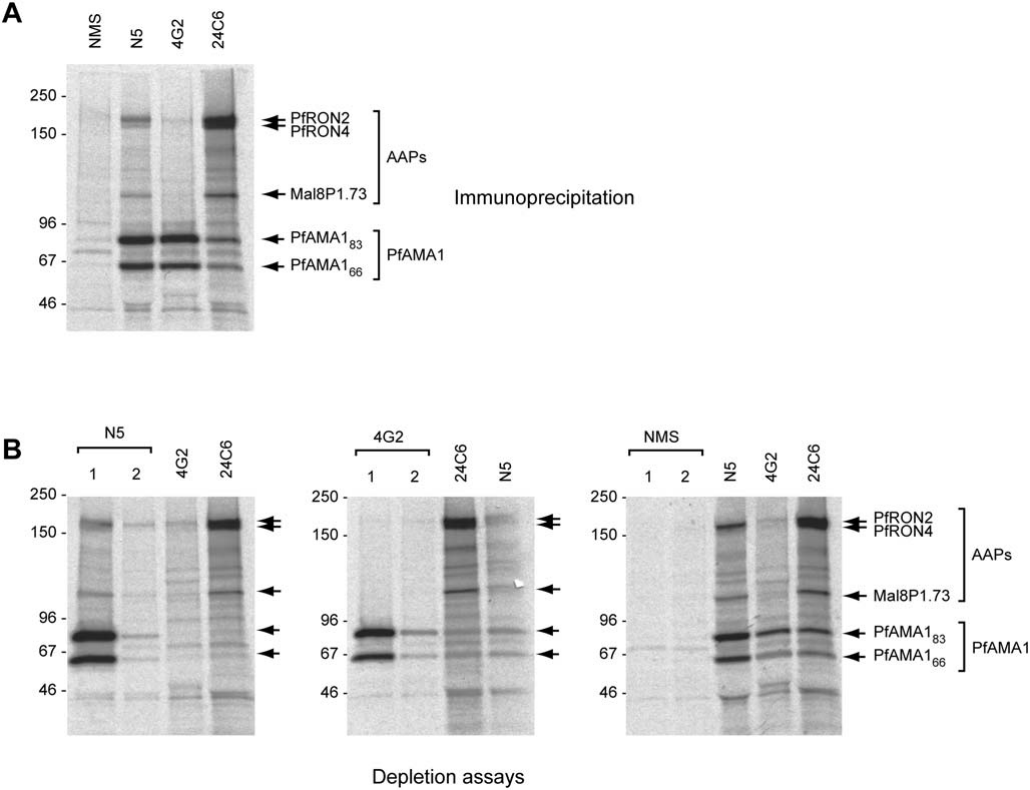
Invasion-inhibitory mAb 4G2 does not bind the PfAMA1-AAP complex. (A). Immunoprecipitation from extracts of biosynthetically radiolabelled schizonts with control normal mouse serum (NMS), polyclonal anti-PfAMA1 serum N5, mAb 4G2 and anti-PfRON4 mAb 24C6. Although mAb 4G2 efficiently precipitated PfAMA1, it did not co-precipitate any of the three AAPs (indicated). These results were completely reproducible in 10 independent immunoprecipitation experiments. (B). Immuno-depletion experiments. Schizont extracts were subjected to two consecutive rounds of depletion (labelled 1 and 2) using either anti-PfAMA1 serum N5, or mAb 4G2, or control normal mouse serum (NMS), followed in each case by IP from the depleted extracts with the reciprocal antibody or mAb 24C6. Each track contains proteins eluted with SDS from the immunoprecipitation matrix. Depletion of the PfAMA1-AAP complex could not be achieved using mAb 4G2, even though it efficiently bound free PfAMA1. In all cases, immunoprecipitated proteins were detected by fluorography. Note that the slight differences in profile at ∼50 kDa in some of the tracks is due to the presence of (unlabelled) immunoglobulin heavy chain on the gels, and the fact that the rat mAb 4G2-derived heavy chain migrates slightly higher on SDS PAGE than that of the mouse antibodies.

We repeated the above IP experiments using mAb 4G2. As shown in Figure 2A, whilst both PfAMA1_83_ and PfAMA1_66_ were immunoprecipitated by mAb 4G2, no AAPs were co-precipitated in this case. This suggested that either mAb 4G2 is unable to recognise the intact complex, binding only free PfAMA1 present in the extracts, or that addition of mAb 4G2 to the schizont extracts disrupts any existing PfAMA1-AAP complex. To distinguish between these possibilities, parasite extracts were subjected to immuno-depletion using either polyclonal serum N5 or mAb 4G2, followed by IP with either the reciprocal antibody or the anti-PfRON4 mAb 24C6. As shown in Figure 2B, N5 depleted virtually all PfAMA1 in the samples, including most of the PfAMA1-AAP complex. In contrast, two rounds of depletion with mAb 4G2 precipitated free PfAMA1 but did not result in any AAP co-precipitation, whereas subsequent IP with either N5 or mAb 24C6 precipitated the PfAMA1-AAP complex. These results indicate that mAb 4G2 does not destabilise the PfAMA1-AAP complex, but specifically cannot bind it. Comparison of the relative amounts of PfAMA1 immunoprecipitated by mAb 4G2 in two rounds of depletion and subsequently by N5, confirmed previous findings in *Toxoplasma* [12],[26] that in the parasite most AMA1 is not associated with the AAPs. It was also interesting to note that, following depletion of essentially all the PfAMA1 with N5, IP with anti-PfRON4 mAb 24C6 resulted in efficient co-precipitation of PfRON2 and Mal8P1.73, indicating that these three AAPs are able to interact in the absence of PfAMA1. Collectively, these results lead to two conclusions: first, they show that the 4G2 epitope cannot be accessed by mAb 4G2 in the intact PfAMA1-AAP complex; and second, they show that the three AAPs can associate with each other independently of PfAMA1.

### Episomally expressed PfAMA1 is correctly trafficked and interacts with AAPs

**Figure 3:**
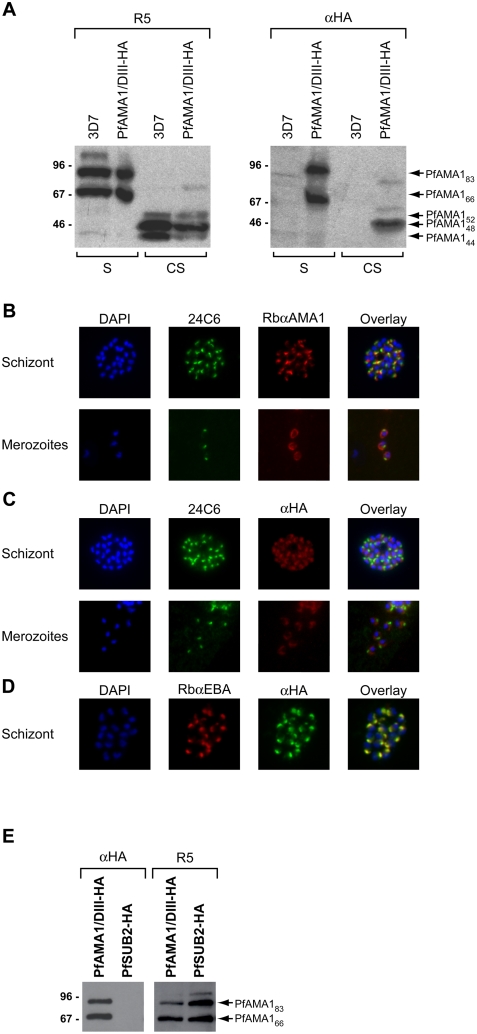
Correct trafficking and post-translational processing of episomally expressed PfAMA1. (A). Western blot of parental 3D7 and transgenic parasite lines harbouring a construct for expression of PfAMA1/DIII-HA. Anti-PfAMA1 serum R5 detected mature and processed forms of PfAMA1 in both parental and transgenic lines, whilst the epitope-tagged protein detected with the anti-HA mAb 3F10 (αHA) was present only in transgenic parasites. S, schizont extracts; CS, culture supernatants. (B)–(D). IFA of the PfAMA1/DIII-HA line probed with the anti-PfRON4 mAb 24C6, a rabbit anti-PfAMA1 polyclonal serum (RbαAMA1), mAb 3F10 (αHA), or a rabbit polyclonal anti-EBA-175 serum (RbαEBA). (E). Proteins immunoprecipitated with anti-PfRON4 mAb 24C6 from extracts of the PfAMA1/DIII-HA and PfSUB2-HA lines, analysed by Western blot with mAb 3F10 (αHA) or anti-PfAMA1 serum R5. Whilst PfAMA1 was efficiently co-precipitated from both extracts, no mAb 3F10-reactive species were co-precipitated from extracts of the PfSUB2-HA clone (note that mature PfSUB2-HA migrates on SDS-PAGE at ∼75 kDa; [5]).

The above results suggested that mAb 4G2 may interact with a region of PfAMA1 involved in AAP complex formation. To investigate this possibility, we adopted a strategy of expression of *pfama1* transgenes in the parasite. We have previously demonstrated, using allelic replacement via homologous recombination, that a synthetic re-codonised *pfama1* gene (*ama-1syn*) can fully complement the function of the endogenous *pfama1* gene [24]. For the current study, a construct was designed to obtain episomal expression of the *ama-1syn* gene, modified by insertion of a haemagglutinin (HA) epitope tag within a loop in domain III of the PfAMA1 ectodomain. Parasites transfected with this plasmid were expected to express the tagged transgene under control of the *pfama1* promoter, on a background of expression of the endogenous allele. To determine whether the transgene product (called PfAMA1/DIII-HA) was correctly trafficked in *P. falciparum*, a parasite line harbouring the construct was analysed by Western blot using polyclonal anti-PfAMA1 antibodies (which recognise both endogenous and episomal gene products) and the anti-HA mAb 3F10. As shown in Figure 3, PfAMA1_83_ and the processed PfAMA1_66_ form were detectable in both transgenic and parental wild-type parasites with polyclonal anti-PfAMA1 serum R5, but were detected only in transgenic parasites with the HA-specific mAb 3F10 (Figure 3A). This indicated that the epitope-tagged PfAMA1/DIII-HA was expressed and correctly processed in the transgenic parasites, in turn suggesting its correct trafficking to the micronemes (where processing to PfAMA1_66_ occurs; [1],[3]). The PfAMA1/DIII-HA product was also correctly shed into culture supernatants following merozoite release (Figure 3A, CS lanes). Interestingly, none of the transgenic protein was shed in the minor PfAMA1_44_ form, likely due to the fact that the HA epitope tag within the domain III loop straddles the internal “nick” site at Asn464, preventing the usual partial cleavage that occurs at this site around the time of PfSUB2-mediated shedding [6]. Correct trafficking of the transgene product was confirmed by IFA using mAb 3F10 (Figure 3B–D); expression of PfAMA1/DIII-HA was detected at the apical prominence of both intracellular and free transgenic merozoites in a pattern identical to that observed with antibodies to PfAMA1 and to another microneme protein, EBA-175, but distinct from that seen with the rhoptry-specific mAb 24C6. Peripheral membrane staining was also observed on free merozoites with mAb 3F10 (Figure 3C, merozoites), confirming that PfAMA1/DIII-HA was correctly translocated from micronemes onto the merozoite surface following schizont rupture.

To determine whether the PfAMA1/DIII-HA gene product is able to interact with endogenous AAPs, transgenic parasites were analysed by IP using the anti-PfRON4 mAb 24C6. PfAMA1/DIII-HA was efficiently co-precipitated (Figure 3E). The specificity of this interaction was confirmed by immunoprecipitation with mAb 24C6 from extracts of a different transgenic *P. falciparum* clone in which the chromosomal PfSUB2 gene has been modified by fusion to a C-terminal triple-HA tag [5]; like PfAMA1, PfSUB2 is trafficked to micronemes and eventually relocated onto the merozoite surface. Immunoprecipitation with mAb 24C6 from this clone did not result in co-precipitation of HA-tagged PfSUB2 (Figure 3E). Collectively, these results show that episomally-expressed PfAMA1/DIII-HA is correctly expressed, trafficked and processed in *P. falciparum*, and that it interacts with PfRON4 in a manner similar to endogenous PfAMA1.

### The base of the PfAMA1 domain II loop does not play a role in interaction with AAPs

**Figure 4:**
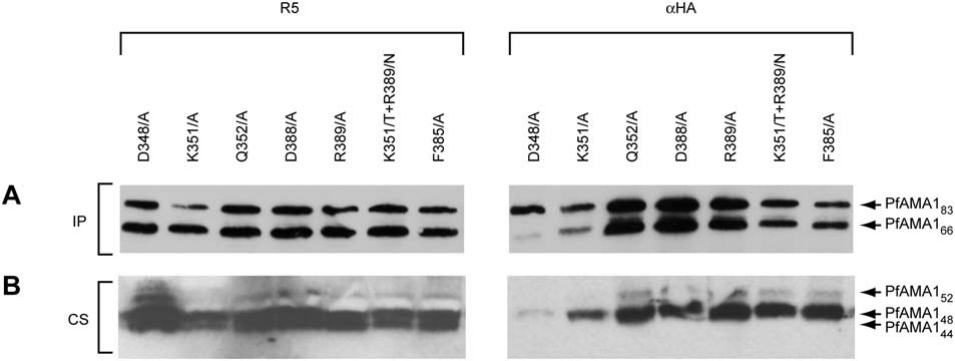
Mutations within the 4G2 epitope do not prevent PfAMA1-PfRON4 interaction. (A). Western blot of immunoprecipitates from parasite lines expressing various mutants of PfAMA1/DIII-HA. IP was performed with the anti-PfRON4 mAb 24C6. (B). Western blot of culture supernatants (CS) collected from the same lines. Blots were probed with either anti-PfAMA1 serum R5 or anti-HA mAb 3F10 (αHA). Parasite lines expressing PfAMA1/DIII-HA and mutants thereof exhibited no discernible growth phenotype (data not shown).

To examine the structural requirements for the interaction between PfAMA1 and the AAPs, we next expressed a range of mutant forms of PfAMA1/DIII-HA in *P. falciparum* and examined their capacity to interact with PfRON4. In view of the above evidence suggesting possible overlap between the mAb 4G2 epitope and the AAP binding site(s) on PfAMA1, we first explored residues directly involved in recognition of PfAMA1 by mAb 4G2. Six mutant constructs were produced, designed to express the single residue alanine substitution mutants PfAMA1/DIII-HA-D348/A, PfAMA1/DIII-HA-K351/A, PfAMA1/DIII-HA-Q352/A, PfAMA1/DIII-HA-D388/A, PfAMA1/DIII-HA-R389/A, and PfAMA1/DIII-HA-F385/A. All these residues have previously been shown to be required for mAb 4G2 binding [17],[24]. An additional construct was also produced for expression of PfAMA1/DIII-HA-K351/T+R389/N, in which two residues were substituted with the corresponding residues from AMA1 of the human malaria parasite *P. vivax*. Mutant constructs were transfected into *P. falciparum* and the resulting transgenic lines examined as above. As shown in Figure 4, all the mutant proteins were expressed and correctly processed (although mutant PfAMA1/DIII-HA-D348/A appeared to be processed to the PfAMA1_66_ form rather inefficiently, perhaps due to the previously noted structurally destabilising effect of this particular mutation [24]). Furthermore all interacted efficiently with PfRON4 as shown by co-precipitation with the anti-PfRON4 mAb 24C6. It was concluded that the individual residues known to be required for binding of mAb 4G2 to PfAMA1 are not essential for formation of the PfAMA1-AAP complex.

### Hydrophobic trough residues are involved in the interaction between PfAMA1 and AAPs

**Figure 5:**
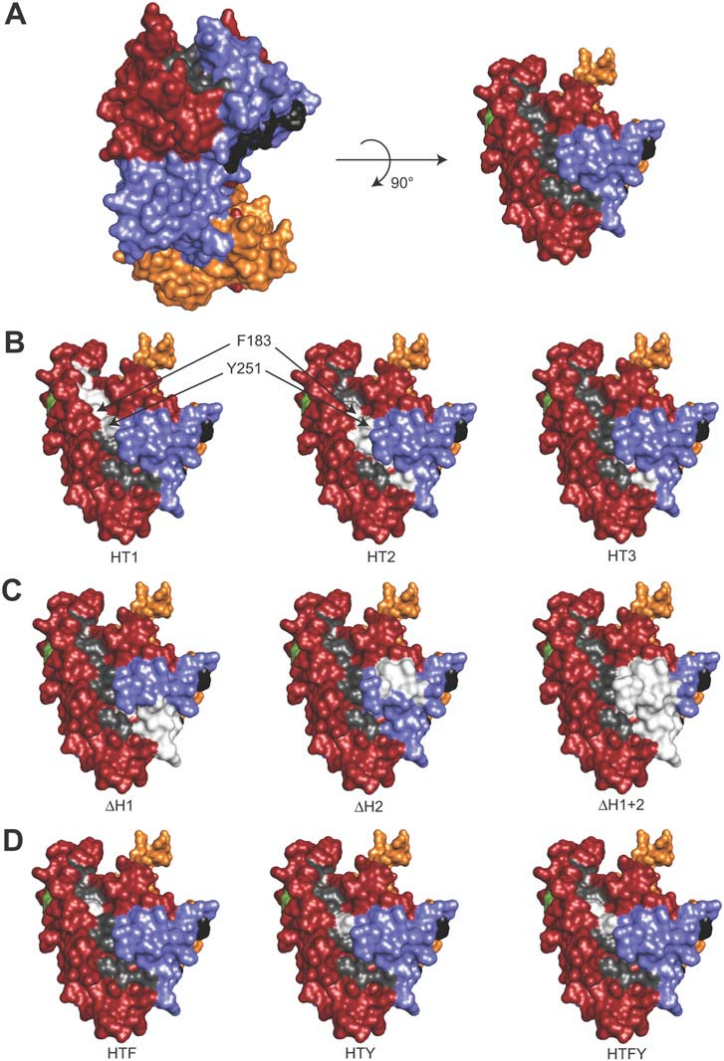
PfAMA1 mutations explored within and flanking the hydrophobic trough. (A). Two views of the molecular surface representation of the PfAMA1 model based on the x-ray crystal structures of PfAMA1 and PvAMA1. Domain I is shown in red, domain II in blue, domain III in mustard, the hydrophobic trough in grey, the 4G2 epitope in black and the mAb 1F9 epitope residue Glu197 in green, visible on the left in the 90° rotated view. (B). Hydrophobic trough substitution mutants. PfAMA1 view as shown in (A) right-hand side, with from left to right mutants PfAMA1/DIII-HA-HT1 (Phe183, Ile190, Tyr251, Tyr202, Met224 in white), PfAMA1/DIII-HA-HT2 (Val169, Phe183, Tyr251, Ile252, Leu357, Phe367 in white) and PfAMA1/DIII-HA-HT3 (Leu357, Phe367 in white). (C). Deletion mutants. PfAMA1 view as shown in (B) showing in white the domain II loop residues removed in mutants PfAMA1/DIII-HA-ΔH1, PfAMA1/DIII-HA-ΔH2 and PfAMA1/III-HA-ΔH1+2. (D). Phe183/Tyr251 substitution mutants. PfAMA1 view as shown in (B) with, from left to right, mutants PfAMA1/DIII-HA-HTF, -HTY and –HTFY. Residues substituted with alanine are in each case shown in white. Figures were created using PyMOL (DeLano Scientific).

**Figure 6:**
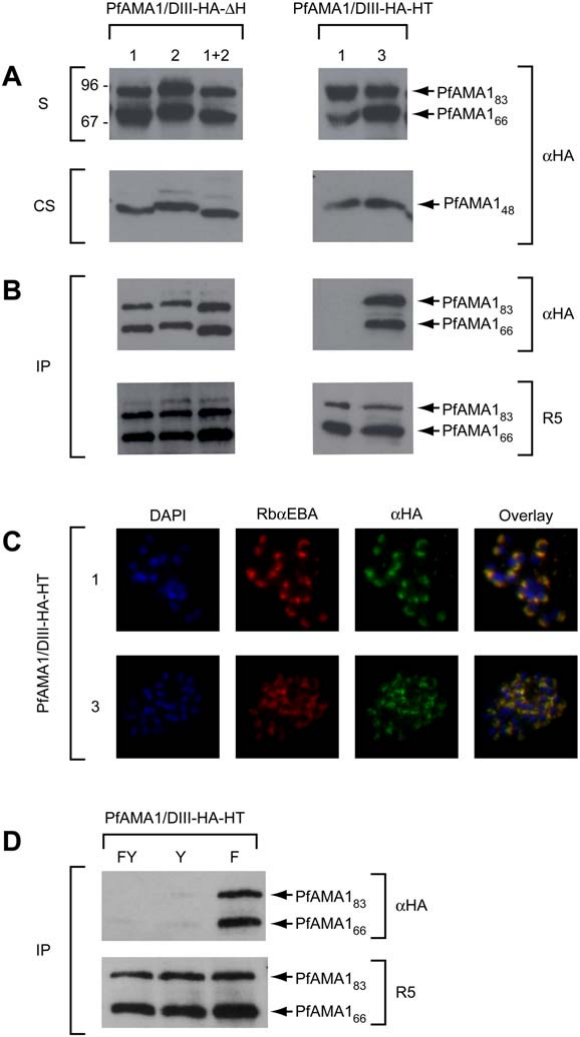
PfAMA1 hydrophobic trough residues are critical for interaction with PfRON4. (A). Western blot of total schizont extracts (S) and culture supernatants (CS) from parasite lines expressing deletion mutants (PfAMA1/DIII-HA-ΔH1, -ΔH2, and ΔH1+2) and hydrophobic trough mutants (PfAMA1/DIII-HA-HT1 and -HT3). Blots were probed with the anti-HA mAb 3F10 (αHA). (B). Western blot of IPs from extracts of the same lines, probed with anti-HA mAb 3F10 (αHA) or anti-PfAMA1 serum R5. IP was performed using anti-PfRON4 mAb 24C6. (C). IFA of schizonts from lines expressing mutants PfAMA1/DIII-HA-HT1 and -HT3, showing identical sub-cellular localisation of the mutant transgene product, detected by mAb 3F10 (αHA), with the microneme marker EBA-175 (RbαEBA). (D). Western blot of IPs from extracts of the Phe183 and Tyr251 mutants (PfAMA1/DIII-HA-HTFY, -HTF and –HTY), probed with anti-HA mAb 3F10 (αHA) or anti-PfAMA1 serum R5. IP was performed using anti-PfRON4 mAb 24C6. Note that all these mutants except PfAMA1/DIII-HA-ΔH1 and -ΔH1+2 (consistent with our previous findings, [24]) were reactive with the anti-PfAMA1 mAb 4G2 (Figure S1) confirming that they were conformationally correct.

Our previous analysis of the mAb 4G2 epitope has shown that it lies adjacent to a conspicuous groove on the surface of domain I of the PfAMA1 ectodomain (Figure 5A). Referred to as the hydrophobic trough, it is made up of residues that are mostly conserved across all *Plasmodium* species examined to date, and are conserved with respect to their hydrophobicity in all apicomplexan AMA1 sequences so far identified [18]. Of particular note, the trough lies adjacent to the mAb 4G2 epitope, raising the possibility that, if it plays a role in AAP binding, binding of AAPs to the trough might interfere with mAb 4G2 binding. Also, on the other side of the trough lies a highly polymorphic residue, Glu197, essential for binding of another, strain-specific invasion-inhibitory mAb called 1F9 [21],[27]. To explore whether the hydrophobic trough plays a role in PfAMA1-AAP complex formation, a further set of constructs were produced to express PfAMA1/DIII-HA mutants possessing substitutions or deletions within or adjacent to the trough. Mutants PfAMA1/DIII-HA-HT1 and PfAMA1/DIII-HA-HT2 possess alanine substitutions of aromatic residues Phe183 and Tyr251 - which lie at the centre of the trough within 6.7 Å of each other – plus substitutions of either residues Ile190, Tyr202 and Met224 (PfAMA1/DIII-HA-HT1; Figure 5B left-hand side) or Val169, Ile252, Leu357 and Phe367 (PfAMA1/DIII-HA-HT2; Figure 5B centre). Mutant PfAMA1/DIII-HA-HT3, in contrast, possessed alanine substitutions of just two conserved residues, Leu357 and Phe367, which lie in the domain II loop and form part of one end of the hydrophobic trough (Figure 5B right-hand side). Three further deletion mutants were produced called PfAMA1/DIII-HA-ΔH1, PfAMA1/DIII-HA-ΔH2, and PfAMA1/DIII-HA-ΔH1+2 (Figure 5C). These lack respectively the first, the second, or both of two stretches, Tyr353-Lys368 and Asp373-Ser377, within the upper part of the domain II loop adjacent to the trough. Constructs for episomal expression of all six mutants were transfected into *P. falciparum* and the resulting transgenic lines examined as previously. Analysis of mutant PfAMA1/DIII-HA-HT2 by IFA showed that it was correctly trafficked, but further examination by immunoprecipitation and Western blot indicated it degraded rapidly following detergent extraction of schizonts, possibly due to destabilisation of the global fold of the molecule (data not shown). As shown in Figure 6, all the other mutant transgene products were correctly expressed and processed, and could be detected by Western blot in extracts or culture supernatants of the lines (Figure 6A). Immunoprecipitation with mAb 24C6 (Figure 6B) showed that all three deletion mutants and PfAMA1/DIII-HA-HT3 were co-precipitated with PfRON4, indicating that they efficiently formed a complex with this AAP. In contrast, mutant PfAMA1/DIII-HA-HT1 did not co-precipitate with PfRON4, even though it was properly trafficked (Figure 6C) and even though endogenous PfAMA1 was efficiently co-precipitated from extracts of this same line. This strongly suggests that one or more of the hydrophobic trough residues Ile190, Tyr202, Met224, Phe183 and Tyr251 are critical for formation of the PfAMA1-AAP complex.

To further dissect this observation, a final trio of mutants possessing alanine substitutions of the two central trough residues Phe183 and Tyr251, either separately (PfAMA1/DIII-HA-HTF and PfAMA1/DIII-HA-HTY), or together (mutant PfAMA1/DIII-HA-HTFY; see Figure 5D), were produced and evaluated in the same way. As with the other mutants shown in Figure 5, these were correctly trafficked and processed (not shown). As shown in Figure 6D, whereas mutation of just Phe183 alone resulted in a product that retained binding to RON4, the two mutants containing the Tyr251Ala substitution were severely impaired in their ability to interact with RON4. Taken together with the absence of binding displayed by mutant PfAMA1/DIII-HA-HT1, these results strongly suggest that the central trough residue Tyr251 (which is completely conserved across all apicomplexan AMA1 sequences) plays a critical role in AAP complex binding. In view of the proximity of this residue to those comprising the 4G2 epitope (less than 30 Å; Figure 5), our results strongly suggest that the inability of mAb 4G2 to recognise the PfAMA1-AAP complex is a result of steric hindrance or local conformational changes introduced into PfAMA1 by the bound AAP complex. Given the large mass of an IgG molecule (∼180 kDa), the converse is also likely to be true: binding of mAb 4G2 to free PfAMA1 is likely to prevent formation of the PfAMA1-AAP complex.

## Discussion

The molecular role of AMA1 has been a subject of considerable investigation, but remains poorly understood. Gene disruption attempts in haploid stages of *Plasmodium* and *Toxoplasma* [8],[28] were important in indicating that AMA1 is essential for parasite viability. More insight into its role has been derived from approaches that more subtly interfere with its function. Using an elegant gene knockdown system in *Toxoplasma*, Mital et al. [26] showed that parasites expressing AMA1 at <0.5% of wild-type levels exhibit a block in the second, intimate attachment step of host cell invasion, as well as a defect in rhoptry secretion. An electron microscopic study on the effects of an invasion-inhibitory mAb specific for AMA1 of the simian malaria species *P. knowlesi* observed that arrested extracellular merozoites had formed initial attachments with the host erythrocyte but not the close range interactions required for junction formation [29]. Together with the revelations of Alexander et al. [12],[13] showing an association with AMA1 with AAPs in the moving junction, these data are all consistent with a role for AMA1 in assembly of this remarkable structure, certainly as a structural component of the junction and possibly also as a mediator of the signal(s) that triggers discharge of RON4 and the other AAPs to form the AMA1-AAP complex. How do the results of the present study contribute to this picture?

Three major conclusions can be drawn from our findings, as follows. First, that the AMA1-AAP complex first identified in *Toxoplasma* is entirely conserved in the human malaria parasite *P. falciparum*. Second, that the intact complex, although present in detergent extracts of mature *P. falciparum* schizonts, is selectively not recognised by the invasion-inhibitory mAb 4G2. Third, that certain residues within the hydrophobic trough, a prominent and conserved feature of the membrane-distal domain I of the molecule, are essential for PfAMA1-AAP complex formation. Some of the mutations we introduced into our episomally-expressed PfAMA1 constructs to investigate the requirements for AAP binding could have introduced far-reaching structural changes into the molecule, necessitating caution in the interpretation of some of our results. As an example of this, the alanine substitutions of Leu357 and Phe367 in mutants PfAMA1/DIII-HA-HT2 and PfAMA1/DIII-HA-HT3 would be expected to have a substantial destabilising effect on the interface between domain I and the domain II loop. However, the ability of PfAMA1/DIII-HA-HT3 to interact with both the AAP complex (Figure 6C) and mAb 4G2 (Figure S1) suggests that the substituted residues are not on their own critical for stabilisation of the domain II loop. On the other hand, the clear absence of RON4 binding displayed by mutants PfAMA1/DIII-HA-HTFY, PfAMA1/DIII-HA-HTY and PfAMA1/DIII-HA-HT1 strongly implicate solvent-exposed hydrophobic trough residues – and specifically the central, completely conserved aromatic residue Tyr251 - as being key players in AAP complex formation. The fact that this trough lies adjacent to a cluster of residues we have previously shown to be required for 4G2 binding strongly suggests that it is the presence of bound AAPs that selectively prevents interaction of mAb 4G2 with the PfAMA1-AAP complex, presumably due to steric hindrance between these large molecules or as a result of local conformational changes introduced into PfAMA1 upon AAP binding. For the reasons laid out below, we propose that the invasion-inhibitory activity of mAb 4G2 is a direct result of this.

Several reports, including our own earlier studies [6],[7], have suggested that AMA1 translocates from micronemes to the parasite surface well before interaction with the host cell surface. For technical reasons (not least the difficulty of isolating invasive *P. falciparum* merozoites) the precise timing of this relative to host cell entry has not been demonstrated conclusively in *P. falciparum*. However, in both *P. knowlesi* and *Toxoplasma*, AMA1 has been clearly demonstrated on the surface of invasive and/or actively invading merozoites and tachyzoites [7]–[10],[12]. The fact that monoclonal and polyclonal antibodies to AMA1 efficiently block invasion in both genera, as well as in the related apicomplexan *Babesia* [11], lends additional support to this, showing that surface-resident parasite AMA1 is accessible to antibody prior to the point of invasion. In contrast, current knowledge of RON4 trafficking suggests that this protein does not relocalise to the parasite surface with the same timing as AMA1. Detailed studies in *Toxoplasma* [12],[15],[25] and our own analyses in *P. falciparum* (not shown, but see e.g. Figure 3B) have shown that RON4 is not detectable in any location other than the rhoptries prior to the point of initiation of invasion, suggesting that its discharge to interact with AMA1 and partake in moving junction formation occurs subsequent to movement of AMA1 onto the parasite surface. Assuming that trafficking of the other AAPs follows a similar pathway to that of RON4 (RON2 is a rhoptry neck protein, and Ts4705/Mal8P1.73 is also likely rhoptry-derived; [30]) our data support the following simple model. Discharge of AMA1 from micronemes onto the parasite surface occurs at some point prior to interaction with the host cell. Anti-AMA1 antibodies are able to immediately bind the solvent-exposed protein. Upon host cell binding, and at or around the point of junction formation, RON4 plus the other components of the AAP complex are discharged from rhoptries, assembling with the surface-resident AMA1 via the hydrophobic trough to participate in formation of the moving junction. If – as in the case of mAb 4G2 - the specificity of the bound antibody is such that it blocks AMA1-AAP assembly, junction formation is impaired and the entire invasion pathway is arrested at that point.

Clearly, one additional way to test the validity of the above model would be to directly demonstrate that binding of mAb 4G2 to free PfAMA1 prevents assembly of the PfAMA1-AAP complex. However, although the schizont extracts used in this study contained free PfAMA1 and free AAP complex as well as pre-formed complex, we were unable to detect any change in the relative abundance of these forms over time. Also, depletion of the free PfAMA1 with mAb 4G2 did not detectably destabilise existing PfAMA1-AAP complex suggesting that, once the complex is formed, the affinity between its constituents is high. On the other hand, despite exhaustive efforts we have been unable to reconstitute the complex *in vitro* using recombinant, correctly folded PfAMA1 ectodomain and parasite-derived AAPs (C. Collins and M. Blackman, unpublished), suggesting that assembly of the complex may require specific conditions that cannot be mimicked in crude detergent extracts. Other models are consistent with our data – for example, it is possible that mAb 4G2 may interfere with a distinct function for PfAMA1 in signal transduction – but we favour the above proposal because of the very clear inability of mAb 4G2 to bind pre-formed PfAMA1-AAP complex. It is worth noting that both the published x-ray crystal structures of AMA1 reveal that segments of the domain II loop show signs of extensive mobility. This is displayed either through weak or absent electron density in the crystal structures, or high temperature factors, whilst in the PvAMA1 structure the loop is very disordered [17],[18]. Thus, in addition to the possibility of simple steric hindrance, one further possibility is that this mobility of the domain II loop could be responsible for a cryptic AAP-binding site that remains hidden until the ligand is present. Binding of mAb 4G2 to the domain II loop could block a conformational change in it that is required for AAP binding, or alternatively might induce a conformational change that is incompatible with AAP binding. Determination of the three-dimensional structures of both the AMA1-AAP complex and the PfAMA1-mAb 4G2 complex is now required to reveal the complete details of how AAP binding interferes with recognition of PfAMA1 by mAb 4G2, and *vice versa*.

Our findings add to a steadily accumulating dataset pinpointing the importance of the hydrophobic trough in AMA1 function. As pointed out by Bai and colleagues [18], the fact that the trough is surrounded by polymorphic residues (including some of the most polymorphic positions in the entire molecule), whilst residues within the trough itself are highly conserved, suggests analogies with receptor binding pockets in viral proteins such as influenza haemagglutinin, where flanking polymorphisms enable antigenic diversification to ‘escape’ neutralising antibody responses [31]. The 4G2 epitope is conserved across all known *P. falciparum* isolates, but polymorphic residues critical for binding of the invasion-inhibitory mAb 1F9 lie in close proximity to the hydrophobic trough (Figure 5) [21],[27]. The functional importance of the trough and its flanking segments is further supported by the recent demonstration that single chain antibodies targeting the trough can prevent invasion [32], and the epitope recognised by a protective P. *yoelii* AMA1-specific mAb localises to the domain II loop [33]. Thus, there are substantial existing data highlighting the hydrophobic trough as a functionally important region of AMA1. By showing that residues within the trough are required for AAP binding, and by demonstrating a link between epitope accessibility and AMA1-AAP complex formation, our study provides the first plausible mechanistic explanation for the invasion-inhibitory activity of antibodies that recognise this region of AMA1

We found it surprising that the majority of the PfAMA1 mutations studied did not discernibly affect binding to PfRON4. These include all the point mutations in residues implicated in 4G2 binding, in particular the Asp348Ala substitution in mutant PfAMA1/DIII-HA-D348/A, which we have previously found to affect the overall fold of PfAMA1 [24]. The fact that these mutants retained their RON4 binding capacity suggests that the surface involved in AAP formation is fairly localised – although it is very likely larger than that encompassed by the single residue substituted in the PfAMA1/DIII-HA-HTY mutant. Furthermore, our observation that the anti-PfRON4 mAb 24C6 co-precipitated both the mature 83 kDa and processed 66 kDa forms of PfAMA1 clearly demonstrates that prosequence removal is not a prerequisite for AAP binding. Our work also raises questions about the topology of the complex. Our observation that the three AAPs can be co-precipitated following depletion of PfAMA1 is consistent with the findings of Lebrun et al. in *Toxoplasma* [15], showing that the AAPs can interact independently of PfAMA1. It is conceivable that the AAPs may traffic from the rhoptries as a pre-formed complex. In the absence of antibody reagents to the other *P. falciparum* AAPs identified here, PfRON2 and Mal8P1.73, we were unable to ascertain whether these proteins can associate individually with PfAMA1 in the absence of PfRON4 binding. Further work is required to establish the association between the various components, in particular the stoichiometry of the interaction(s) and identification of the partner(s) that interacts directly with PfAMA1. In this regard, it may be significant that in their study showing an interaction between RON4 and PfAMA1, Alexander et al. [13] did not observe any co-precipitation of any other AAPs. The discrepancy between their results and our own may be a result of the different conditions used for immunoprecipitation (e.g. their use of RIPA buffer, which contains deoxycholate and SDS in addition to NP40), but whatever the case their data suggest that at least RON4 can directly associate with PfAMA1. Finally, whilst this manuscript was under peer review, Cao and colleagues [34] published a study showing formation of a complex between PfAMA1, PfRON4 and PfRON2 in mature schizonts, and confirming localisation of PfRON2 to the rhoptry neck.

There is an increasingly urgent need for an effective antimalarial vaccine, particularly against *P. falciparum*. It has long been recognised that not all antibodies against AMA1 possess invasion-inhibitory properties [19]. If interfering with assembly of the PfAMA1-AAP complex is a common mechanism by which invasion-inhibitory antibodies function, our observations may inform the rational design of optimised sub-unit constructs that specifically direct the humoral immune response towards the production of antibodies targeting the PfAMA1-AAP interface. Alternatively, it may be possible to identify small compounds that bind the interface with high affinity and similarly block this critical step in invasion.

## Materials and Methods

### Antibodies

Anti-PfAMA1 antisera were produced as described previously by immunisation with recombinant PfAMA1 ectodomain (Ile97-Lys546) expressed in *Pichia pastoris* using a synthetic codon-optimised synthetic gene called *ama-1syn* [17],[24],[35]. Mouse serum N5 and rabbit serum Rb1 were raised by immunisation with native recombinant protein, whilst mouse serum R5 was raised by immunisation with reduced and alkylated protein as previously described [17],[24]. The HA-specific mAb 3F10 (Roche) was used for detection of HA-tagged proteins. Protein G Sepharose (GE Healthcare) was used to purify rat mAb 4G2, a kind gift of Alan Thomas (Biomedical Primate Research Centre, Rijswijk, The Netherlands), from hybridoma culture supernatants. The PfRON4-specific mouse mAb 24C6 [25] was a kind gift of Jean-François Dubremetz, University of Montpellier 2, France.

### Construction of episomal expression plasmids

For episomal expression of PfAMA1 transgenes in *P. falciparum*, a single HA epitope tag (YPYDVPDYA in single letter amino-acid code; [36]) was introduced into a region of *ama-1syn* encoding a loop in domain III. Structural analyses (not shown) making use of the three-dimensional structure of PfAMA1 [17],[18] indicated that a tag at this location within the ectodomain would not affect overall protein conformation. To introduce the tag, sequence encoding 459KRIKLNDND467 in construct pST2A-sgPfa1 [24] was modified to encode the HA tag by Quikchange site-directed mutagenesis (Stratagene), resulting in construct pST2A-sgA1/DIII-HA. To produce a construct for episomal expression of the full-length HA-tagged protein (PfAMA1-DIII-HA) in *P. falciparum* under control of the authentic *ama1* promoter, sequence spanning the HA tag was excised using *Stu*I and *Age*I (Roche) and cloned into pHAM-sgPfa1/HA [24] restricted with the same enzymes, giving rise to plasmid pHAM-sgA1/DIII-HA. For expression of various mutants of PfAMA1-DIII-HA, appropriate mutations were introduced into pST2A-sgPfa1 by Quikchange site-directed mutagenesis, and sequence encompassing these mutations sub-cloned by restriction with *Pst*I and *Nco*I into pHAM-sgA1/DIII-HA. Nucleotide sequences of all cloned products were confirmed by sequencing on both strands.

### Parasite culture and transfection

Blood stages of *P. falciparum* clone 3D7 were maintained and synchronized in medium containing the serum substitute Albumax using standard procedures [37]. Ring stage parasites at 5–10% parasitaemia were transfected by electroporation with 70–100 µg of plasmid DNA as described previously [5]. Selection for lines harboring the input plasmid was carried out using 10 nM WR99210 (Jacobus Pharmaceuticals, New Jersey, USA). Synchronous schizont preparations were frozen directly for IP analysis or saponin-treated and analysed by Western blot. For analysis of proteins released into culture supernatants, purified schizonts were cultured for 8 h in medium without Albumax in the presence of fresh human erythrocytes to allow reinvasion and shedding of invasion proteins. Following clarification through a 0.2 µm filter (Vivascience), culture supernatants were concentrated 50-fold using a 10 kDa molecular mass cut-off Vivaspin 6 column (Vivascience) before analysis by Western blot.

### Immunoprecipitation, SDS-PAGE and Western blot

Cultures containing highly mature, synchronous schizonts were metabolically labelled for 1 h in methionine-free medium containing 100 µCi ml^−1^ L-[^35^S]-methionine and L-[^35^S]-cysteine Promix™ (GE Healthcare) as described previously [38]. Parasites were washed and cultured for a further hour before being harvested by centrifugation and storage at −80°C. Immunoprecipitation from Nonidet P40 extracts of the radiolabelled schizonts was as described previously [38] using anti-PfAMA1 serum N5, mAb 4G2, mAb 24C6 or normal mouse serum (NMS) as control. Immunoprecipitated proteins were solubilised in SDS sample buffer and subjected to SDS-PAGE under non-reducing or reducing conditions followed by either fluorography or transfer to Hybond-C extra nitrocellulose membrane (GE Healthcare). Membranes were probed with mAbs or polyclonal antibodies as described previously [38].

### Proteolytic digestion and MALDI-TOF mass spectrometry

Immunoprecipitated samples were reduced and alkylated, fractionated by SDS-PAGE, subjected to in-gel tryptic digestion and digests analysed by MALDI-TOF mass spectrometry, all as previously described [4].

### Indirect immunofluorescence analysis (IFA)

Thin films of *P. falciparum* cultures containing mature schizonts and naturally-released free merozoites were air-dried, fixed in 4% (w/v) formaldehyde for 30 minutes (Agar Scientific Ltd.), permeabilized for 10 minutes in 0.1% (w/v) Triton ×100 and blocked overnight in 3% (w/v) bovine serum albumin in PBS. Slides were probed with mAbs and polyclonal sera as described previously [5], mounted in Citifluor (Citifluor Ltd., Canterbury, U.K.), and images collected using AxioVision 3.1 software on an Axioplan 2 Imaging system (Zeiss) using a Plan-APOCHROMAT 100×/1.4 oil immersion objective.

### Molecular modelling of the PfAMA1 ectodomain

Coordinates for PfAMA1 domains I and II (PDB ID code 1Z40 and 2H86; [18]), and the complete PvAMA1 ectodomain (PDB ID code 1W81 and 1W8K; [17]), which lacks electron density for much of the domain II loop, were aligned and used to produce an energy minimised model extending from residue 96 to 533, as described previously [24]. Figures were created using PyMOL (DeLano, W.L. The PyMOL Molecular Graphics System, 2002; DeLano Scientific, San Carlos, CA, USA. http://www.pymol.org)

## Supporting Information

Figure S1 Mutations within and flanking the hydrophobic trough of PfAMA1 do not disrupt the overall conformation of the molecule
(0.19 MB PDF)

Table S1 Peptides identified by MALDI-TOF analysis of RON4 tryptic digests
(0.07 MB PDF)

Table S2 Peptides identified by MALDI-TOF analysis of PfRON2 tryptic digests
(0.12 MB PDF)

Table S3 Peptides identified by MALDI-TOF analysis of Mal8P1.73 tryptic digests
(0.10 MB PDF)
