# Supplementary material for: An Inhibitory Antibody Blocks Interactions between Components of the Malarial Invasion Machinery
Source: PLoS Pathog. 2009 Jan 23;5(1):e1000273. doi: 10.1371/journal.ppat.1000273 (PMC2621342; doi:10.1371/journal.ppat.1000273)

Supplementary Figure S1: Mutations within and flanking the hydrophobic trough of PfAMA1 do not disrupt the overall conformation of the molecule.

Western blot of immunoprecipitates from extracts of transgenic parasite lines expressing hydrophobic trough mutants of PfAMA1/DIII-HA. IPs were performed with mAb 4G2 and the Westerns were probed with either polyclonal antibody R5 (to detect both endogenous and transgene-derived PfAMA1), or the anti-HA mAb 3F10 (to detect transgene-derived PfAMA1 only). The results confirm that all the hydrophobic trough mutants except PfAMA1/DIII-HA-ΔH1 and -ΔH1+2 were able to interact with mAb 4G2.

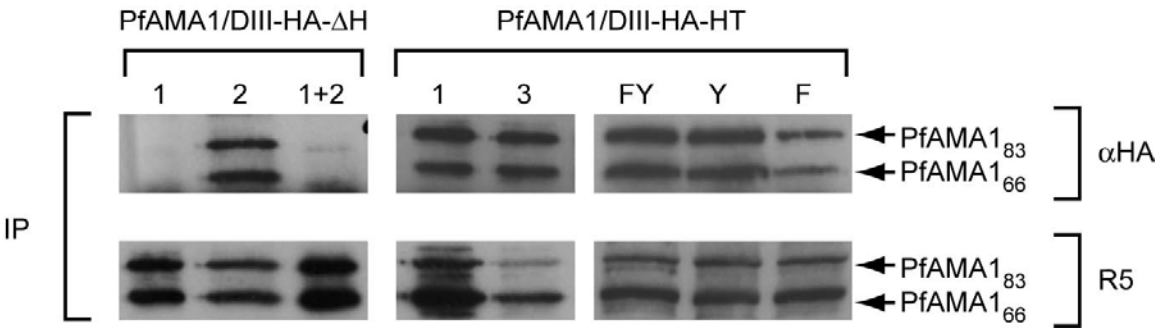

Supplement: Figure S1 — Mutations within and flanking the hydrophobic trough of PfAMA1 do not disrupt the overall conformation of the molecule (0.19 MB PDF) [file ppat.1000273.s001.pdf]
