# Supplementary material for: An Inhibitory Antibody Blocks Interactions between Components of the Malarial Invasion Machinery
Source: PLoS Pathog. 2009 Jan 23;5(1):e1000273. doi: 10.1371/journal.ppat.1000273 (PMC2621342; doi:10.1371/journal.ppat.1000273)
Supplement: Table S1 — Peptides identified by MALDI-TOF analysis of RON4 tryptic digests (0.07 MB PDF) [file ppat.1000273.s002.pdf]

Supplementary Table S1: Peptides identified by MALDI-TOF analysis of PfRON4 tryptic digests

**Peptide sequence**	**Residue numbers**	**Calculated m/z**	**Observed m/z**	**Error +/- ppm**
KLGVSYSR	2695 - 2702	908.508	909.529	14.7
FYETLGIK	2670 - 2677	969.517	970.507	-18.7
GLHVSLRER	2585 - 2593	1065.604	1066.612	-0.1
LALVPFQGIK	2386 - 2395	1084.664	1085.655	-15.6
NMNNNHAQR	2541 - 2549	1097.478	1098.509	20.3
NMNNNHAQR (Met oxidised)	2541 - 2549	1113.473	1114.487	5.7
IIIEIMESAK	2594 - 2603	1145.637	1146.658	12.2
MVLQYLVHLK	2934 - 2943	1242.716	1243.745	17.6
HMVGEHVPPQK	2462 - 2472	1257.629	1258.661	19.5
HMVGEHVPPQK (Met oxidised)	2462 - 2472	1273.624	1274.668	28.7
INSYFHYTEK	2874 - 2883	1300.609	1301.650	26.0
DSGKLFMEALEK	2617 - 2628	1366.680	1367.725	27.1
VSSVPFNYENVK	2834 - 2845	1381.688	1382.725	21.3
ERIIIEIMESAK	2592 - 2603	1430.780	1431.836	33.2
CAIKDIIISISSK	2714 - 2726	1446.812	1447.843	16.2
KPSSSIIGSLGNLIK	2846 - 2860	1512.887	1513.929	22.0
YSQNKDYVETLEK	2802 - 2814	1615.773	1616.792	6.9
DHAYLGLIDELLVMNK (Met oxidised)	2785 - 2800	1858.950	1859.912	-24.3
IISVCTLLHLTDMLYK	2893 - 2908	1919.026	1920.034	0.1
IISVCTLLHLTDMLYK (Met oxidised)	2893 - 2908	1935.021	1936.020	-4.5
ILTEMSFYEDSKFYETLGIK	2658 - 2677	2413.176	2414.187	1.4
EGPIITPLEGEQAGTAHKEDVTHK	2438 - 2461	2556.282	2557.290	0.1
KMIANNDNYTCSVYQNSNCSNK (Met oxidised)	1329 - 1350	2640.100	2641.175	25.4
NTEENKIEEQYNNINHHSHDINK	2008 - 2030	2819.286	2820.245	-17.3
