# Supplementary material for: An Inhibitory Antibody Blocks Interactions between Components of the Malarial Invasion Machinery
Source: PLoS Pathog. 2009 Jan 23;5(1):e1000273. doi: 10.1371/journal.ppat.1000273 (PMC2621342; doi:10.1371/journal.ppat.1000273)
Supplement: Table S2 — Peptides identified by MALDI-TOF analysis of PfRON2 tryptic digests (0.12 MB PDF) [file ppat.1000273.s003.pdf]

Supplementary Table S2: Peptides identified by MALDI-TOF analysis of PfRON2 tryptic digests

Peptide sequence	Residue numbers	Calculated m/z	Observed m/z	Error +/- ppm
EPFGVAR	1833 - 1839	774.402	775.382	-36.8
KAFDLLR	1290 - 1296	861.507	862.472	-49.8
GFEEYKR	1622 - 1628	927.445	928.420	-35.8
TTTFYGER	1192 - 1199	973.450	974.422	-37.1
ELIYGDIR	1338 - 1345	977.518	978.482	-45.0
DIGPNKSER	408 - 416	1014.509	1015.483	-33.9
SYYDVYGR	417 - 424	1021.450	1022.425	-32.4
YASPTYDPK	36 - 44	1040.481	1041.569	76.6
DGYLSESER	823 - 831	1054.457	1055.496	29.8
NPNIFPGSPR	551 - 560	1097.562	1098.532	-34.0
YIYNDFMK (Met oxidised)	1357 - 1364	1108.490	1109.466	-28.8
YAELSHENR	1850 - 1858	1117.515	1118.501	-19.8
HNFSQYDSK	1586 - 1594	1124.489	1125.463	-30.1
YTLWYEPR	1330 - 1337	1126.545	1127.534	-16.7
LQDLQLEER	1664 - 1672	1142.593	1143.564	-32.1
ILSLGNMLLR (Met oxidised)	1758 - 1767	1144.664	1145.630	-36.5
YTLWYEPR (Trp oxidised)	1330 - 1337	1158.535	1159.527	-13.3
GSYHYISYAK	2177 - 2186	1187.561	1188.552	-14.4
VQFLNSTSSIR	1409 - 1419	1250.662	1251.683	10.3
FANSTSMLIPHK (Met oxidised)	1511 - 1522	1360.681	1361.689	0.6
IDDKDFVHNFK	1908 - 1918	1376.672	1377.671	-6.5
GNYIENINNIAR	1460 - 1471	1389.700	1390.726	12.7
LQDLQLEEREK	1664 - 1674	1399.731	1400.756	12.3
SFLPSNEIELEK	936 - 947	1404.714	1405.702	-13.9
DITFIPIESRPK	1179 - 1190	1414.782	1415.807	12.0
WDQFINKELVR	1689 - 1699	1446.762	1447.793	16.1
DIGAGPVASCFTTR	2028 - 2041	1450.687	1451.713	12.4
CEFEESSLYNPK	1262 - 1273	1501.639	1502.639	-5.7
EYDDNKYNPYNK	425 - 436	1561.668	1562.694	11.1
IGDYLNISFEYSR	970 - 982	1575.757	1576.773	5.1
VEHALWFGATIDIKK (Trp oxidised)	1773 - 1787	1758.930	1759.898	-22.6
HLLENNIDNNIIFSR	1727 - 1741	1810.933	1811.904	-20.1
LYYNSLALGELVEPIR	1077 - 1092	1848.998	1849.963	-23.6
LLQHIPAHMLENITSSIR	1548 - 1565	2072.120	2073.047	-39.2
LLQHIPAHMLENITSSIR (Met oxidised)	1548 - 1565	2088.115	2089.059	-30.6
EYLCHLFVNNPGTKPLER	1059 - 1076	2186.094	2187.006	-43.8
HLTNNTDDTILHMIENAEK (Met oxidised)	1000 - 1018	2224.043	2224.970	-36.1
ENSLLFNQSDKWDQFINK (Trp oxidised)	1678 - 1695	2257.065	2257.970	-45.4
GTYGNNETPYYVEHPEYDNGK	498 - 518	2446.035	2446.927	-47.1
VVEDSKHLLENNIDNNIIFSR	1721 - 1741	2468.266	2469.136	-55.9
VNINITNNGTHGNPHNNNSYGHK	257 - 279	2515.170	2516.053	-49.7
ILKEYLCHLFVNNPGTKPLER	1056 - 1076	2540.357	2541.211	-60.6
GTKVIFYMPGNEQGVIPNNIQNK	46 - 68	2560.311	2561.053	-103.8
NQDNNSNKYNNEQNGGYESDGNSPNSR	230 - 256	3015.221	3015.926	-100.6
