# Supplementary material for: An Inhibitory Antibody Blocks Interactions between Components of the Malarial Invasion Machinery
Source: PLoS Pathog. 2009 Jan 23;5(1):e1000273. doi: 10.1371/journal.ppat.1000273 (PMC2621342; doi:10.1371/journal.ppat.1000273)
Supplement: Table S3 — Peptides identified by MALDI-TOF analysis of Mal8P1.73 tryptic digests (0.10 MB PDF) [file ppat.1000273.s004.pdf]

Supplementary Table S3: Peptides identified by MALDI-TOF analysis of Mal8P1.73 tryptic digests

Peptide sequence	Residue numbers	Calculated m/z	Observed m/z	Error +/- ppm
LLGLISR	775 - 781	770.501	771.484	-32.4
NVGFLLR	552 - 558	817.481	818.458	-37.2
CDHFNR	823 - 828	847.339	848.309	-45.4
WFNYNK	559 - 564	870.402	871.334	-88.1
IIMQIIR	650 - 656	885.547	886.507	-53.7
IIMQIIR (Met oxidised)	650 - 656	901.542	902.483	-73.5
NMGLEHER	1094 - 1101	984.445	985.390	-63.0
IAPFNFFR	472 - 479	1010.534	1011.481	-60.0
LLMYYYR	396 - 402	1020.510	1021.455	-61.5
LLMYYYR (Met oxidised)	396 - 402	1036.505	1037.434	-76.4
TQISLFIHK	928 - 936	1085.623	1086.551	-73.9
CKPTKISIR	783 - 791	1101.633	1102.608	-29.5
GIGNEIIVHTR	1204 - 1214	1207.667	1208.632	-35.9
NKINYEIFVK	840 - 849	1266.697	1267.632	-57.8
IVLGCIHDYGGR	759 - 770	1358.676	1359.660	-17.7
GLDDSLYTFKSK	333 - 344	1372.687	1373.644	-37.5
GIGNEIIVHTRIP	1204 - 1216	1417.804	1418.779	-23.1
NVDSIDVMFEPR	58 - 69	1420.666	1421.642	-22.3
NVDSIDVMFEPR (Met oxidised)	58 - 69	1436.660	1437.625	-29.8
LMSSLNDPVDILR	802 - 814	1471.770	1472.745	-22.5
ESLATMHSLDIIR	147 - 159	1484.766	1485.744	-20.1
LMSSLNDPVDILR (Met oxidised)	802 - 814	1487.765	1488.728	-30.0
TGNESIFAIDDIIK	486 - 499	1534.788	1535.738	-37.3
ELERTQISLFIHK	924 - 936	1612.894	1613.840	-38.1
IVLGCIHDYGGREK	759 - 772	1615.814	1616.781	-25.0
TDKIVLGCIHDYGGR	756 - 770	1702.846	1703.777	-45.0
IAPFNFFREETQEK	472 - 485	1754.863	1755.789	-46.5
QLDSQDISSLNNFKR	126 - 140	1763.880	1764.803	-48.3
FFENIKNEMINMSSK	108 - 122	1830.864	1831.775	-53.0
NYNDLVNFSEVDYLLR	625 - 640	1972.953	1973.863	-49.7
ASEVLKESLATMHSLDIIR	141 - 159	2112.125	2113.003	-61.5
ASEVLKESLATMHSLDIIR (Met oxidised)	141 - 159	2128.120	2129.019	-50.9
HLPSDVNGNSLANTVNFVILNK	1148 - 1169	2365.239	2366.140	-45.1
VSEFDYAIQNPEANIIVFDGNNYISSYALR	1064 - 1093	3421.647	3422.184	-137.3
